# Small Pox

**Published:** 1883-02

**Authors:** 


**SMALL-POX.**—"Poc" is an old English word, meaning a pouch, pocket, or bag. "Pocs," means more than one, is its plural, and for convenience is spelled Pox, from the many little pits or pouches made in the skin by this disease. The Latin name is "variola," which means a pimple. A person who had the small-pox milked a cow, and the pox appeared on her teats; this cow was milked by a girl, when the pox appeared on her hands, but she did not seem to mind it. A good many other milkers had the same appearances, but all went about their business as if nothing was the matter with them.

Nearly a hundred years ago, a young man was in a drug-store near Bristol, England, when a dairy-maid called for some advice. Small-pox was prevailing at the time, and she was asked by the young clerk if she was not afraid of catching it? She replied: "I can't take it, because I've had the cow-pox." In an instant the thought flitted across the mind of the youth, if small-pox was communicated to a cow by man, and the cow could in turn communicate it back to a man, but with the difference that when thus re-communicated, it was not only divested of its horrors, but fortified the person against taking the small-pox, as seemed to have been an impression which had grown up among the milkmaids, then it might be the case that cow-pox could be given to a man artificially, by taking matter from the poc of a cow and introducing it into the system of a man, just as small-pox was given to man artificially, by matter from a poc on a man. This idea was at first vague and unfixed in words, but when in the practice of years, young Jenner (for this was the apothecary's boy) observed that he always failed to give small-pox by inoculation to the milkmaids who had taken the cow-pox, he determined to try the experiment of vaccination, that is, giving cow-pox to a man by the matter from a cow, and thus rendering him insusceptible of the terrible small-pox from any amount of exposure. This experiment was successfully made on his eldest son, in November, 1789. But it was not until May, 1796, that a decisive experiment was made to ascertain if the matter from a person having cow-pox would give cow-pox to a human being. James Phipps, aged eight years, was vaccinated with matter taken from the hands of Sarah Nelmes. He passed through the disorder in a manner perfectly satisfactory, and in July following, all efforts made to give him small-pox by inoculation with small-pox matter, failed to take effect. So that in 1796, vaccination as a preventive of small-pox was demonstrated; and at a very opportune time, too; for three quarters of a million of persons were perishing with small-pox every year. In Prussia alone, forty thousand persons died of small-pox annually. After vaccination was introduced, only three thousand small-pox deaths took place in one year; while Sweden, Denmark, and Germany, where vaccination was systematically performed on the whole population, remained absolutely free from small-pox for twenty years, when the people, having grown remiss in the performance of vaccination, scattering cases of small-pox began to appear again. So great was the boon to the world considered, that the British Parliament, in 1802, gave Jenner fifty thousand dollars, and in 1807 voted him a hundred thousand dollars more. Jenner died at his native place, in great honor, in 1823, in his seventy-fifth year.

The matter of small-pox impregnates the air immediately around the person or bedding of the patient; and any unvaccinated individual, or one who has not had the small-pox, who comes within ten feet of such person or the bedding, is very sure to be attacked with small-pox, and to have the pimples appear within a fortnight.

In some cases vaccination wears out, and ceases to be a protection against small-pox, and exposure to it gives varioloid.

The longer a person remains free from small-pox after vaccination, the more severe the attack will be, if it is taken at all.

Those vaccinated in infancy are most liable to have varioloid between the ages of sixteen and twenty-five. This being so, a most important practical inference is to be drawn, that the occurrence of puberty in some way diminishes the power of vaccination against infection; hence it becomes the imperative duty of every parent to have the child vaccinated on entering the fifteenth year. If it does not take, no harm has been done; if it does take, the chances of an odious and fearful disease have been with great certainty removed. This re-vaccination should be repeated at twenty-five, most especially if that at fifteen did not take.

**Pickles.**—"An excellent way to make pickles which will keep a year or more is—drop them into boiling hot water, but not boil them; let them stay ten minutes, wipe them dry, then drop them into cold spiced vinegar. They will not need to be put into salt and water."

